# The role of circulating anti-aging αKlotho in cardiac aging

**DOI:** 10.46439/signaling.3.057

**Published:** 2025

**Authors:** Dong I Lee, Dao-Fu Dai

**Affiliations:** 1 Division of Cardiology, Department of Medicine, Johns Hopkins University School of Medicine, Baltimore, MD, USA; 2 Department of Pathology, Johns Hopkins University School of Medicine, Baltimore MD, USA

## Abstract

Aging contributes significantly to the deterioration of cardiac function and increases the prevalence of heart failure, including those with reduced or preserved ejection fraction. Heart failure with preserved ejection fraction (HFpEF) is highly prevalent in the elderly population and it has become a leading cause of morbidity and mortality in this group. This commentary discusses the important findings and broader implications of the study by Daneshgar *et al*. on the role of the anti-aging hormone α-Klotho in alleviating diastolic dysfunction in the aged heart via Sirtuin1 (Sirt1)-mediated pathways. Using aged and Klotho-deficient mouse models, they demonstrated that soluble Klotho (sKL) supplementation improved cardiac diastolic function, reduced left ventricular hypertrophy and fibrosis, and increased capillary density. Mechanistically, the cardioprotective effects of sKL were found to rely on Sirt1-mediated regulation of DNA damage pathways and cardiac protein acetylation. These findings provide new insights into the therapeutic potential of targeting the Klotho-Sirt1 axis for HFpEF and other age-related cardiovascular diseases.

## Commentary

Aging is an inevitable biological process that significantly affects various organs, including the heart. Cardiac aging, with its associated structural and functional changes, can lead to left ventricular (LV) hypertrophy, diastolic dysfunction, increased arterial stiffness, and reduced overall cardiac functional reserve [1,2]. As life expectancy increases worldwide, the prevalence of heart failure, particularly HFpEF, accounts for nearly half of all heart failure cases. HFpEF is a symptomatic heart failure characterized by impaired diastolic function due to a reduced ability of the heart to relax and fill with blood while maintaining a normal ejection fraction. Despite its prevalence, the mechanisms underlying HFpEF remain poorly understood and, unlike heart failure with reduced ejection fraction (HFrEF), treatment options for HFpEF are limited. Age-related alterations in critical cardiac proteins, oxidative stress and mitochondrial dysfunction are some of the factors thought to contribute to HFpEF [3,4], although many other factors related to cardiometabolic stress, obesity, kidney dysfunction, pulmonary hypertension and inflammation have been implicated in HFpEF. In this commentary we will focus on aging as a major risk factor for HFpEF.

First identified as an anti-aging hormone predominantly expressed in the kidney, αKlotho has emerged as a promising therapeutic candidate due to its systemic effects on cellular aging, inflammation and oxidative stress. It exists in two primary forms: a transmembrane protein that acts as a co-receptor for fibroblast growth factor 23 (FGF23) and a soluble form that is released into the circulation. The soluble or secreted klotho (sKL), a circulating form of Klotho, exerts systemic effects on multiple organs and regulates multiple signaling pathways [5–9]. Previous studies have shown that Klotho deficiency accelerates age-related phenotypes, including cardiovascular disease, neurodegeneration, and renal dysfunction. Conversely, increased Klotho expression has been associated with improved outcomes in age-related diseases, including reduced oxidative stress, improved endothelial function, and enhanced tissue repair mechanisms [10,11]. In the cardiovascular system, Klotho has shown potential protective effects by alleviating vascular calcification and maintaining vascular elasticity. However, its direct effects on cardiac function, particularly in HFpEF, have not yet been fully explored. Prior studies suggested that Klotho may improve diastolic dysfunction by reducing inflammation and fibrosis in cardiac tissues [12–14]. Recent studies have also highlighted the potential neuroprotective role of Klotho in Alzheimer’s disease and other neurodegenerative conditions by modulating synaptic plasticity and reducing amyloid beta deposition [15,16]. In addition, Klotho has been shown to play an important role in glucose metabolism and may benefit the treatment of diabetes and metabolic syndrome [9,17]. In this study, sKL treatment significantly improved diastolic dysfunction and capillary rarefaction in aged wild-type and Klotho-deficient mice, supporting its potential translational role in ameliorating diastolic dysfunction in HFpEF patients during aging. However, further research is needed to confirm these findings.

Sirtuin1 (Sirt1), a nicotinamide adenine dinucleotide (NAD^+^)-dependent deacetylase, is a key regulator of cellular processes related to metabolism, oxidative stress responses, DNA repair, and aging [18]. Sirt1 expression declines with age and contributes to several age-related diseases, including cardiac dysfunction. The main mechanism involves deacetylation of target proteins, including transcription factors and enzymes important for cellular homeostasis. Sirt1 activity is highly dependent on cellular NAD^+^, a molecule essential for energy production and DNA repair, linking it to metabolic status and mitochondrial function. Sirt1 promotes mitochondrial biogenesis and function by deacetylating and activating peroxisome proliferator-activated receptor gamma coactivator 1-α (PGC-1α), a transcriptional coactivator essential for mitochondrial gene expression. The decline in Sirt1 activity with age contributes to reduced mitochondrial efficiency, leading to increased production of reactive oxygen species (ROS) and oxidative stress [19–22]. Sirt1 facilitates DNA repair by deacetylating proteins involved in double-strand break repair, such as Ku70, demonstrating that its function is critical in aging cells where DNA damage accumulates over time and exacerbates cellular dysfunction [18]. Sirt1 exerts anti-inflammatory effects by deacetylating the Rel A/p65 subunit of NF-κB, thereby suppressing the transcription of pro-inflammatory genes, which are relevant in the aging heart where chronic low-grade inflammation contributes to cardiac dysfunction. Previous studies have shown that reduced Sirt1 levels in aging hearts are associated with impaired cardiac function, increased fibrosis, and diminished angiogenesis [23–25]. Pharmacological or genetic activation of Sirt1 has been shown to attenuate age-related cardiac decline in experimental models. In this study, the authors demonstrated that sKL supplementation restores Sirt1 expression in aged hearts. The cardioprotective effects of Sirt1 were highlighted by its role in the deacetylation and inactivation of checkpoint kinase-2 (CHK2). CHK2 is a critical “checkpoint” protein in the cell cycle, responsible for monitoring cell health and linking to DNA damage response. By deacetylating and inactivating CHK2, Sirt1 helps maintain cellular health and provides protective effects on heart cells [26]. These findings highlight the central role of Sirt1 in mediating the cardioprotective effects of sKL.

The interaction between Klotho and Sirt1 represents a promising therapeutic target for mitigating age-related diseases, particularly in the cardiovascular system. Both proteins act synergistically to maintain mitochondrial function, suppress inflammation, and enhance cellular stress responses. The interplay between Klotho and Sirt1 is therefore a key aspect of the study. In aged hearts, reduced Sirt1 expression leads to mitochondrial dysfunction, increased oxidative stress and impaired cardiac function. The Klotho-Sirt1 axis plays a critical role in reducing NF-κB-mediated inflammation, a key feature of aging and chronic diseases, by suppressing pro-inflammatory cytokines. This anti-inflammatory effect helps maintain tissue integrity and function [18,21,25,27]. Recent research by Wu *et al.* has shown that Klotho can directly upregulate Sirt1 transcription in vascular endothelial cells, protecting against endothelial dysfunction and atherosclerosis [24]. This study demonstrated that sKL supplementation restores Sirt1 expression in aged hearts, leading to reduced CHK2 acetylation and amelioration of diastolic dysfunction. This interaction highlights the synergistic roles of Klotho and Sirt1 in alleviating age-related cardiac phenotypes . Future studies focusing on this axis may provide novel interventions to delay aging and improve longevity.

**Figure 1.**
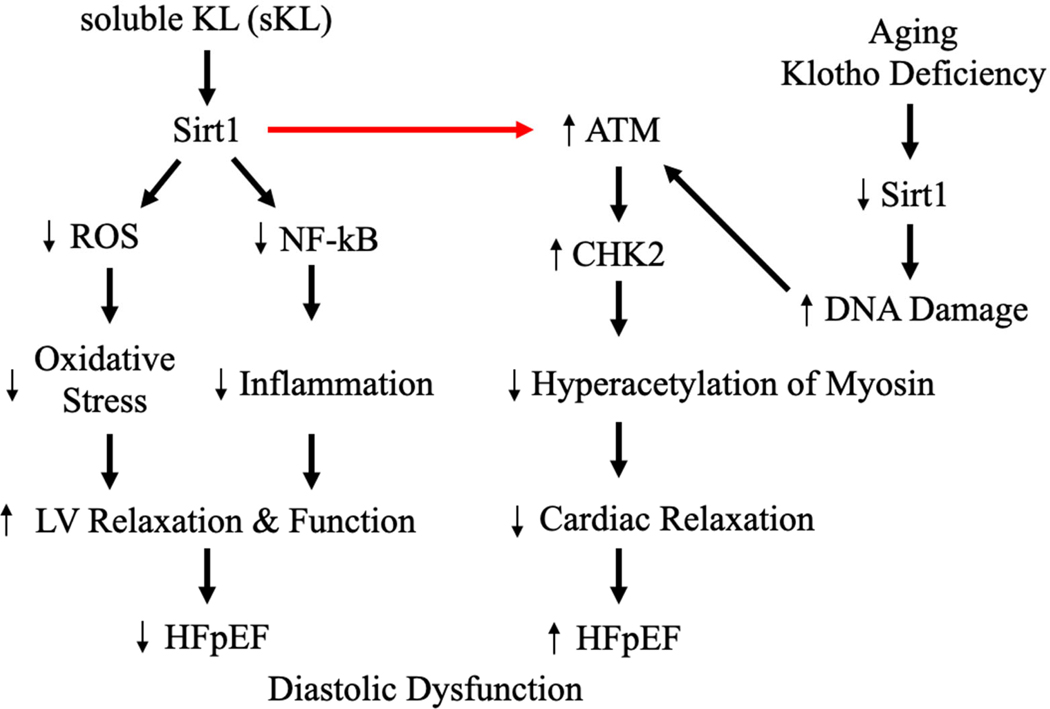
Soluble Klotho and Sirt1 pathway ameliorating heart failure.

One of the most novel findings from this study published in *GeroScience* is the interaction between Klotho and Sirt1, suggesting that Klotho may stabilize Sirt1 expression by reducing ubiquitin-mediated degradation, although the exact mechanism remains to be investigated [13] (Figure 1). The researchers utilized wild-type and heterozygous Klotho-deficient mice to access the effects of 10 weeks of sKL supplementation on left ventricular hypertrophy (LVH), diastolic dysfunction, and other age-related cardiac phenotypes. They demonstrated that Klotho-deficient mice exhibited exacerbated age-related LVH and diastolic dysfunction, which were significantly ameliorated by sKL administration. For example, echocardiographic parameters such as the ratio of early diastolic mitral annular velocity to atrial contraction velocity (E’/A’, Tissue Doppler) improved in sKL-treated mice, indicating enhanced diastolic function. Histological analyses further revealed reduced fibrosis and increased capillary density in sKL-treated hearts, emphasizing the multiple cardioprotective effects of Klotho. The decreased capillary rarefaction in the aged heart, which was aggravated by klotho deficiency, suggests that coronary microvascular dysfunction may play a critical role in HFpEF.

Importantly, the study shed light on the underlying mechanisms, focusing on the Sirt1 pathway. Sirt1, a cardioprotective protein known to regulate DNA damage responses and mitochondrial function, was found to be depleted in aged and Klotho-deficient hearts. sKL supplementation restored Sirt1 expression, which in turn suppressed the DNA damage response pathway mediated by ataxia-telangiectasia mutated (ATM) kinase and CHK2. These results suggest that Klotho alleviates diastolic dysfunction by modulating the Sirt1-CHK2. Furthermore, proteomics and acetylomic analyses, which study changes in proteins and chemicals, have provided insights into post-translational modifications associated with aging and Klotho deficiency. Aged and Klotho-deficient hearts exhibited hyperacetylation of critical sarcomeric proteins, such as β-myosin heavy chain (encoded by MYH7), which impairs ventricular relaxation, contributing to diastolic dysfunction. This evidence positions the Klotho-Sirt1 axis as a critical therapeutic target for addressing cardiac aging.

The methods used in the study are well designed and appropriate to address the research questions. The use of echocardiography and intracardiac pressure measurements provides an accurate assessment of cardiac function (including thorough examinations of diastolic function), and the exercise intolerance by Treadmill and the presence of lung congestions confirmed the presence of heart failure. The acetylation proteomics provides insight into potential novel molecular changes underlying the observed physiological effects, complementary to prior reports of total proteomics. Although the study provides compelling evidence in mouse models, it is not yet known whether these findings can be applied to humans, but the current study provides a scientific basis to consider the therapeutic potential of Klotho for the treatment of age-related HFpEF. One limitation is that the current study only observed a 10-week treatment period, and this is relatively short in the context of aging. It would be valuable to investigate whether long-term Klotho supplementation has sustained benefits or potential side effects. Furthermore, while the authors highlight the importance of the Sirt1 pathway, other pathways, such as NRF2 or FGF23, may also play an important role in mediating the effects of Klotho [28]. Further studies are needed to delineate the full spectrum of Klotho’s cardioprotective mechanisms.

The results of this study highlight the significant potential of sKL supplementation as a novel therapeutic strategy for HFpEF and other age-related cardiovascular diseases. By targeting the Klotho-Sirt1 axis, sKL may attenuate diastolic dysfunction, capillary rarefaction, and DNA damage, thereby improving cardiac function and resistance to aging. Whether combining sKL with Sirt1 activators, which are already being investigated for other age-related diseases, may have synergistic benefit remains unclear. Notably, the study sheds light on the role of protein hyperacetylation in heart failure, an understudied mechanism, and suggests that modulating acetylation status may open new avenues for cardiac-specific therapies. As Sirt1 deacetylation depends on NAD^+^, restoring age-related decline in NAD^+^ has also been shown promising anti-aging effects [29–31]. Beyond cardiovascular health, the systemic effects of sKL, particularly its influence on the Sirt1 pathway, suggest broader applications in the treatment of age-related diseases such as chronic kidney disease and neurodegeneration. However, further rigorous testing is needed to ensure the safety and efficacy of sKL-based therapies in clinical settings.

While the study provides promising preclinical evidence for the therapeutic potential of sKL, several critical areas require further investigation to enable clinical translation of sKL as a therapeutic agent for HFpEF and other age-related diseases. The short duration of sKL supplementation in this study is a critical factor to consider when evaluating its potential long-term effects and safety. Given the limited duration, the ability to assess sustained Klotho supplementation’s full impact remains constrained. Specifically, the long-term effects, including possible side effects or unintended consequences, are not fully understood. One important area of concern is the potential impact of prolonged sKL exposure on organ-specific interactions, particularly in organs involved in Klotho metabolism, such as kidneys. Since Klotho plays a crucial role in kidney function and other critical systems, the safety profile of chronic sKL administration, especially over extended periods, requires careful scrutiny. Long-term studies should focus on understanding how sustained exposure to sKL could alter organ functions, induce dysregulated pathways, or affect aging-related comorbidities, particularly in vulnerable populations like the elderly. In addition, future research may explore any possible issues related to long-term exposure, such as tolerance, or dose-dependent side effects. Given that the current study only addresses short-term supplementation, future study may need to evaluate the potential risks and benefits of prolonged Klotho supplementation. Furthermore, the pathways involving NRF2 and FGF23 warrant more detailed exploration, particularly related to its cardioprotective mechanisms [28,29]. NRF2, a key regulator of oxidative stress, could synergize with Klotho’s antioxidant properties, while FGF23, a known regulator of phosphate metabolism, might modulate Klotho’s effects on mineral metabolism and cardiovascular function in the context of aging. Further investigation into these pathways will be essential for elucidating the full therapeutic potential of Klotho. Another important research gap lies in the hyperacetylation of myosin heavy chain proteins observed in this study, which underscores the need for further studies on protein acetylation in other age-related diseases, such as sarcopenia or muscular dystrophy. Additionally, understanding the systemic effects of Klotho on noncardiac tissues, including the vasculature and kidney, will provide a holistic perspective on its benefits.

Incorporating sKL with existing therapies may also enhance its clinical potential. For example, combining sKL supplementation with Sirt1 activators or other cardioprotective agents could offer a more targeted, synergistic approach for treating HFpEF and other age-related diseases. The development of small molecule mimetics targeting the Klotho-Sirt1 pathway could provide scalable, cost-effective therapies, while the identification of biomarkers to monitor sKL activity will be essential steps towards successful clinical translation. However, translating these findings from mouse models to humans presents significant challenges due to the greater complexity of human aging, which is influenced by a wider range of genetic, environmental, and lifestyle factors [32]. These factors must be carefully considered in future studies to ensure that the insights gained from animal models can be effectively applied to human populations. Therefore, when moving forward with clinical trials, it is crucial to account for the differences between species to optimize the translation of therapeutic strategies.

In conclusion, this study provides compelling evidence that sKL supplementation attenuates age-related diastolic dysfunction through Sirt1-mediated pathways. By reducing cardiac hypertrophy, fibrosis, protein hyperacetylation, and molecular markers of aging, sKL restores both diastolic function and exercise tolerance in aged and Klotho-deficient models. These findings highlight the critical role of the Klotho-Sirt1 axis in preserving cardiac health during aging, positioning sKL as a promising therapeutic candidate for HFpEF, a condition for which there are currently no effective treatments. Moreover, these results extend beyond cardiovascular health, suggesting that Klotho-based therapies may hold broader potential in mitigating age-related diseases and improving overall healthspan. While further translational research is needed to validate these findings in clinical settings, this study represents a crucial step in advancing our understanding of the mechanisms of cardiac aging and paves the way for Klotho-based therapeutic strategies that could enhance the quality of life for the aging population across multiple age-related diseases.
